# GATA-3 expression and its correlation with prognostic factors and survival in canine mammary tumors

**DOI:** 10.3389/fvets.2023.1179808

**Published:** 2023-07-06

**Authors:** Gabriel Saraiva Diniz-Gonçalves, Anna Hielm-Björkman, Vanessa Bonfim da Silva, Lorena Gabriela Rocha Ribeiro, Carlos Humberto da Costa Vieira-Filho, Laís Pereira Silva, Stella Maria Barrouin-Melo, Geovanni Dantas Cassali, Karine Araújo Damasceno, Alessandra Estrela-Lima

**Affiliations:** ^1^Research Center on Mammary Oncology NPqOM/HOSPMEV, Federal University of Bahia, Salvador, Brazil; ^2^DogRisk Research Group, Department of Equine and Small Animal Medicine, Faculty of Veterinary Medicine, University of Helsinki, Helsinki, Finland; ^3^Laboratory of Animal Pathology, Department of Veterinary Medicine, Federal University of Sergipe, São Cristóvão, Brazil; ^4^Department of Veterinary Anatomy, Pathology and Clinics, School of Veterinary Medicine and Zootechny, Federal University of Bahia, Salvador, Brazil; ^5^Laboratory of Comparative Pathology, Department of General Pathology, Federal University of Minas Gerais, Belo Horizonte, Brazil; ^6^Experimental Pathology Laboratory (LAPEX), Gonçalo Moniz Institute, Oswaldo Cruz Foundation (FIOCRUZ), Salvador, Brazil

**Keywords:** breast cancer, transcription factor, prognostic biomarker, tumor progression, survival, canine mammary tumors

## Abstract

**Introduction:**

The transcription factor GATA-3 plays a significant role in mammary gland development and differentiation. Recent studies on human oncology have demonstrated its association with favorable pathologic factors in breast cancer. Canine mammary tumours, proposed as comparative and translational study models, have epidemiological, clinical, biological, and genetic characteristics similar to those of human breast cancers.

**Methods:**

Here, we evaluated the frequency of GATA-3 expression in mammary tumors of dogs and its relationship with prognostic factors and survival. Tumor samples were obtained from 40 female dogs and grouped according to histological type into benign tumors (*n* = 10), carcinoma in mixed tumors (CMTs) (*n* = 20), and aggressive tumors (*n* = 10). CMTs were further separated according to histological grade, and data on clinical staging and diagnosis, histopathological grading, and survival rate were collected.

**Results:**

GATA-3 and estrogen receptor (ER) expression were higher in benign and well-differentiated carcinomas than in aggressive tumors, which showed greater Ki-67 expression. The expression rate of ER in the studied groups was equivalent to that of GATA-3. We identified a strong positive correlation between GATA-3 and ER expression frequencies and a negative correlation between those of GATA-3 and Ki-67. There were associations between GATA-3 (*p* < 0.001), Ki-67 (*p* = 0.003), tumor size (*p* < 0.001), clinical stage (*p* = 0.002), lymph node metastasis (*p* < 0.001), and histological grade (*p* < 0.001) by univariate survival analysis. The parameters ER (*p* = 0.015) and GATA-3 (*p* = 0.005) also influenced survival in a multifactorial manner.

**Discussion:**

Kaplan–Meier analysis of survival curves validated our previous findings that dogs with GATA-3 expression in ≥79.4% of cells had significantly higher survival rates (*p* < 0.001). The performance analysis showed that the expression of GATA-3 in ≥79.4% of cells effectively predicted survival or death in dogs with mammary tumors. Collectively, these results suggest that GATA-3 can be a relevant marker in the study of mammary tumor progression and has potential as a prognosis marker for predicting outcomes in canine mammary tumors.

## 1. Introduction

The identification of molecular markers or biomarkers that can predict the behavior of tumors is particularly important in human breast cancer and canine mammary tumor research, given the variability of the clinical progression of this disease (1–6). GATA-3 is a binding protein belonging to the family of transcription factors that binds to the DNA consensus sequence (A/T) GATA (A/G) and prevails among studied biomarkers; its gene lies on chromosome 10p15 in humans (7, 8). GATA-3 is expressed in the mammary parenchyma and mainly acts in the proliferation and differentiation of luminal epithelial cells that cover the ductal structures of the breast (9). As a marker, absent or low GATA-3 expression in either, human breast cancer (7) and murine models (10) of luminal breast cancer, indicate a loss of cell differentiation, propensity for invasive growth, and development of distant metastases (11).

GATA-3 acts as a tumor suppressor in breast cancers of both transgenic mice and women, since it prevents the epithelial-to-mesenchymal transition induced by TGF-β and components of the TGF-pathway (10, 12–16). Most human mammary tumors originate from luminal epithelial cells, and this protein controls cell differentiation and proliferation of neoplastic cells (10, 14, 17, 18). GATA-3 also alters the tumor microenvironment, as it interferes with angiogenesis, affects macrophages and lymphocytes within intratumoral inflammatory infiltrates, and acts in the regulation of the extracellular matrix via microRNA-29b, thereby inducing the expression of microRNA-29b both directly (binding to the GATA sites in the promoter) and indirectly (inhibiting the TGF-β and NF-κB pathways) (10, 15, 16).

GATA-3 may also promote tumorigenesis through the estrogen receptor (ER), by ER-dependent and ER-independent mechanisms, suggesting that other pathways could influence its expression and function (19, 20). Dysregulation of genes BCL-2 (B-cell CLL/lymphoma 2), DACH1 (Dachshund1), and THSD4 (Thrombospondin, type I domain containing 4) occur in the main processes of cancer progression that are controlled by GATA-3 (20). A recent study reported a weak and positive correlation between the expression of GATA-3 and greater mitotic activity in bladder cancer in dogs. This result suggested that increased GATA-3 expression indicated a worse prognosis for this tumor; however, those authors considered that the stage of tumor progression in the sample set studied was exceptionally homogeneous and emphasized that the biological role of GATA-3 in bladder urothelial carcinoma is still poorly understood (21).

Spontaneous mammary tumors in female dogs are considered models for the study of breast cancer in women (1, 2, 22–25). Additionally, such tumors are frequent among the canine population, and more than 50% of cases are malignant (1, 6, 25, 26), considering that mammary carcinoma in mixed tumors (CMT) has variable malignancy potential depending on histological grade (1). Nevertheless, there are few studies related to the expression of GATA-3 in mammary neoplasms of female dogs.

To the knowledge of the present authors, only two studies have been focused on evaluating the expression or the epigenetics of the GATA-3 transcription factor in canine mammary tumors (27, 28). One study reported on the use of GATA-3 to verify the site of tumor origin in two male dogs with triple-negative mammary carcinoma (27) by employing its reported high sensitivity and specificity as a marker to identify primary and metastatic invasive breast carcinomas (29–31). Triple-negative breast cancer is aggressive in both women and dogs and is characterized by the absence of positivity to hormone receptors (estrogen and progesterone) and human epidermal growth factor receptor-2 (ERBB2/HER2). This tumor can be classified as basal or non-basal depending on the presence or absence of the expression of basal cytokeratins (CK5/6, 17 and CK CK14), respectively. The second study used primary cell cultures of spontaneous canine mammary tumors to characterize cancer-associated molecules, including GATA-3 (28). The results demonstrated that complex adenoma or simple carcinoma cells exhibited lower levels of GATA-3 expression, while considerably higher levels were expressed in complex or mixed carcinoma cells.

Thus, the aims of this study were to evaluate the expression of GATA-3 in spontaneous mammary tumors of female dogs according to the tumor biological behavior, degree of malignancy, and tumor progression in relation to the classic prognoses, as well as to verify the potential of GATA-3 as an independent prognostic factor.

## 2. Materials and methods

### 2.1. Ethical approval

The School of Veterinary Medicine of the Federal University of Bahia (permit number 17/2021) approved this research protocol. All procedures complied with the guidelines of the Brazilian College of Animal Experimentation (COBEA).

### 2.2. Study design and tumor samples

This non-randomized retrospective study included 40 samples of mammary tumors from female dogs assisted by the Nucleus for Research in Mammary Oncology at the Federal University of Bahia, Brazil, selected between 2019 to 2021. The cases were recruited according to the following inclusion criteria: (#1) biopsy from mastectomy, (#2) availability of tissue samples in paraffin blocks for immunohistochemistry, and (#3) clinical information. Clinical and pathological data were obtained from admission and monitoring records. Parameters evaluated included tumor size, clinical staging, presence of metastasis, diagnosis and histopathological grade and survival time. The overall survival time, expressed in days, was defined as the time between the surgical excision of the primary tumor and the date of death or end of this study (December 2021). The survival rate was classified as low when the survival values were ≤365 days and high when the survival values were >365 days; the percentage of dogs that were still alive at the end of the study was determined.

The cases were divided into four groups, each one with ten dogs: group of benign tumors (G-BT), composed by adenomas and benign mixed tumors; group carcinomas in a mixed tumor (CMT) grade I (G-CMT-I); group of CMT grade II (G-CMT-II). The group of aggressive tumours was composed by five samples of solid carcinomas, two samples of pleomorphic lobular carcinomas, which are a variant of invasive lobular carcinoma (32, 33), one sample of micropapillary carcinoma and two CMT grade III.

### 2.3. Histological classification and histological grading

The original hematoxylin–eosin (HE) slides for each case were retrieved and reviewed by two pathologists, blindly and independently, without the prior diagnosis. When necessary new histological sections were obtained from the original paraffin blocks and were stained by the HE method. The histopathological classifications followed the criteria proposed by Goldschmidt et al. (32) and were standardized by Cassali et al. (33). The tumors were graded using the Nottingham System (34) which evaluates the percentage of tubule formation, nuclear pleomorphism and the mitotic index. The areas of invasiveness by the CMTs were used to classify them into Grade I, II, or III (35). Any discrepancies were resolved through a multiheaded microscope by discussion to reach a consensus. Finally, the cases with diagnosis and graduation confirmed by the two evaluators were inserted into the study.

### 2.4. Antibodies and immunohistochemistry

To perform immunohistochemical studies, the following monoclonal antibodies (mAbs) were used to detect: GATA-3, ER, and Ki-67. The biomarkers were assessed according to REMARK criteria for reporting studies on tumour markers (36). Sections (4 μm) were cut from one representative block of each tumor sample. Tissue sections were deparaffinized in xylene and examined using the NovoLink Max Polymer Detection System (Leica Biosystems1). GATA-3 (1:250, clone L50-823, Cell Marque, United States), ER (1:100, clone 1D5, Dako, Carpinteria, United States), and Ki-67 (1:100, clone MIB-1, Dako, Carpinteria, United States) were subjected to heat-induced antigen retrieval with sodium citrate buffer (pH 6.0) in a water bath at 96°C for 30 min. Endogenous peroxidase activity was blocked with 3% hydrogen peroxidase in methanol. Slides were covered with anti-GATA-3 and anti-Ki-67 primary antibodies and incubated at room temperature for 60 min; with the anti-ER as the primary antibody, slides were incubated overnight at 4°C. A polymeric system was used for antibody detection (Novolink^™^ Max Polymer Detection System, Leica Biosystems, Buffalo Grove, IL). Finally, diaminobenzidine (DAB) was used as a chromogen, and sections were counterstained with Mayer’s hematoxylin, dehydrated, and mounted in a synthetic medium. Negative controls were prepared by replacing the primary antibody with normal serum. Samples of previously tested dog mammary glands were used as positive controls.

For quantification of the nuclear expression of GATA-3, ER, and Ki-67, the software ImageJ (National Institutes of Health, Bethesda, Maryland, United States) was used and in all groups, only the neoplastic epithelial cells were considered in the count. Ten random fields were photographed for each case, excluding the necrosis or intense cell density areas, and 1,000 neoplastic cell nuclei were quantified, with or without marking for each antibody. GATA-3, ER, and Ki-67 staining were positive when cell nuclei presented a diffuse nuclear staining pattern. The expression was analyzed quantitatively, and its value was expressed as the percentage of positively stained cells calculated by counting 1,000 cells per section (400 × magnification). The cut-off ≥10% and ≥14% of stained nuclei were considered to classify the case as presenting a positive expression of ER (33) and a high rate of cell proliferation (37), respectively.

### 2.5. Performance indexes of GATA-3 expression

The receiver operating characteristic curve (ROC curve) (38) was used to select the best cut-off value for GATA-3 expression to discriminate distinct evolution to death or survival. The performance analysis included the global accuracy analysis, which was evaluated by the area under the ROC curve (AUC) proposed by Swets et al. (39). The formulae used were: Co-positivity (Co-pos) = [true positives/(true positive samples + false negative samples)] × 100; Co-negativity (Co-neg) = [true negatives/(true negative samples + false positive samples)] × 100; Positive predictive value (PPV) = (true positive samples/total of positive samples) × 100; Negative predictive value (NPV) = (true negative samples/total of negative samples) × 100; Positive Likelihood ratio (LR+) = Co-positivity/(1 − Co-negativity); Negative Likelihood ratio (LR^−^) = (1 − Co-positivity)/(Co-negativity).

### 2.6. Statistical analysis

The data were grouped as follows: tumor size (<5 cm or ≥5 cm), node metastasis (no or yes), clinical staging (v), histological grade (I, II, or III), ER (<10% and ≥10%), Ki-67 (<14% and ≥14%), and survival (≤365 days or >365 days). The survival time was classified as low when the survival values were ≤365 days. Statistical analysis, linear by linear association, was used to compare the relevance between category variables of mammary cancer. Initially, the Kolmogorov–Smirnov test was applied to evaluate the normality of data distribution. Student’s *t*-tests were used for the variables with normal distribution. The Kruskal–Wallis test followed by Dunn’s test was used for variables without normal distribution. The Spearman test investigated possible correlations. The correlation coefficients (*r*) were interpreted according to Pett et al. (40), dividing the classifications into weak (0–0.29), low (0.3–0.49), moderate (0.5–0.69), strong (0.7–0.89), or very strong (0.9–1.0); whether they are positive or negative. Survival curves were generated by the Kaplan–Meier estimation method and compared by Log-rank Mantel-Cox or Cox proportional hazards tests in univariate or multivariate analysis, respectively. The survival analysis was restricted to the 30 female dogs with malignant mammary tumors. The analyses were performed using GraphPad Prism 8.0.2 (GraphPad, San Diego, CA, United States) and SPSS 21 (SPSS Inc., Chicago, IL, United States), and MedCalc for Windows version 19.1.7 (MedCalc Software, Ostend, Belgium). All analyses were conducted using 0.05 as the critical probability level for type I error.

## 3. Results

### 3.1. Prognostic and survival factors

The results of the analyzed prognostic factors and survival in each study group are presented in Table 1. The benign tumor group (G-BT) was composed predominantly of dogs with tumors smaller than 3 cm (9/10) (*p* < 0.001), and all dogs (10/10) in this group were alive at the end of the study and had no history of new nodules. The G-CMT-I group encompassed animals with tumors frequently between 3 and 5 cm (6/10) and no lymph node metastasis; in this group, stages I and II were predominant. 80% of animals (8/10) showed survival classified as high, and 50% (5/10) were still alive at the end of this study (*p* < 0.001). The G-CMT-II group showed a higher frequency of tumors larger than 5 cm (5/10,) and lymph node metastasis occurred in two cases (2/10) (*p* < 0.001). Among the G-AT/CMT-III dogs, there was a majority of large nodules (greater than 5 cm; in 9/10 dogs), lymph node metastasis (8/10), and a predominance of stage IV (8/10); survival was low (≤365 days post-surgery) in most cases (9/10), and all individuals of this group succumbed within 13 months (*p* < 0.001).

**Table 1:** Clinical and pathological characteristics of female dogs with mammary tumors according to the analysed group.

Parameters	Total *n* = 40	Benign Tumors *n* = 10	CMT I *n* = 10	CMT II *n* = 10	Aggressive tumors *n* = 10	Value of *p*
Size						
<3 cm	32.5% (13/40)	90% (9/10)	30% (3/10)	10% (1/10)	0% (0/10)	<0.001^*^
3–5 cm	30% (12/40)	10% (1/10)	60% (6/10)	40% (4/10)	10% (1/10)	
>5 cm	37.5% (15/40)	0 (0/10)	10% (1/10)	50% (5/10)	90% (9/10)	
LN metastasis						
No	66.7% (20/30)	–	100% (10/10)	80% (8/10)	20% (2/10)	<0.001^*^
Yes	33.3 (10/30)	–	0 (0/10)	20% (2/10)	80% (8/10)	
Distant metastasis						
No	100% (30/30)	–	100% (10/10)	100% (10/10)	100% (10/10)	–
Yes	0 (0/10)		0 (0/10)	0 (0/10)	0 (0/10)	
Clinical stage						
Stage I	13,3%	–	30% (3/10)	10% (1/10)	0 (0/10)	<0.001^*^
Stage II	(4/30)	–	60% (6/10)	0 (0/10)	0 (0/10)	
Stage III	20% (6/30)	–	10% (1/10)	70% (7/10)	20% (2/10)	
Stage IV	33.3% (10/30)	–	0 (0/10)	20% (2/10)	80% (8/10)	
Histological grading						
Grade I	33.3% (10/30)	–	100% (10/10)	0 (0/10)	0 (0/10)	<0.001^*^
Grade II	46.7% (14/30)	–	0 (0/10)	100 (10/10)	40% (4/10)	
Grade III	20% (6/30)	–	0 (0/10)	0 (0/10)	60% (6/10)	
Survival rate						
≤365 days	45% (18/40)	80% (8/10)	80% (8/10)	50% (5/10)	90% (9/10)	<0.001^*^
>365 days	55% (22/40)	20% (2/10)	20% (2/10)	50% (5/10)	10% (1/10)	<0.001^*^
% of living dogs	50% (20/40)	100% (10/10)	50% (5/10)	50% (5/10)	0 (0/10)	–

^*^Significant differences (*p* < 0.005); Statistical tests: Exact Fisher Test and Linear by Linear Association. Benign tumors are not staged or graded. G-BT, group of benign tumors; G-CMT-I, group carcinomas in a mixed tumor (CMT) grade I; G-CMT-II, group of CMT grade II; G-AT/CMT-III, group of aggressive tumors and CMT grade III.

### 3.2. Immunohistochemistry

**Figure 1:**
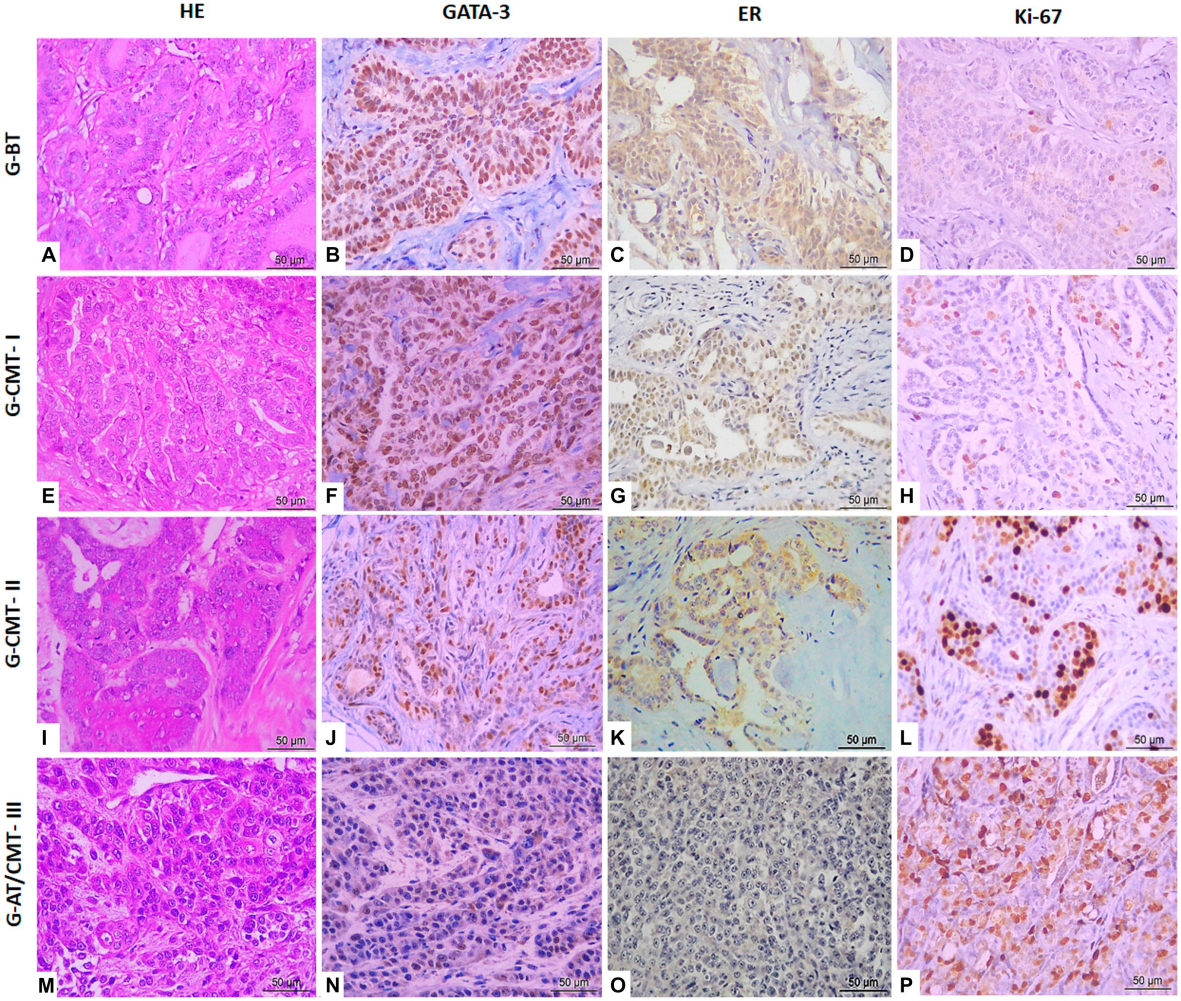
Immunohistochemical expression of GATA-3, ER, and Ki-67 markers in canine mammary cancer samples (benign tumor, CMT grade I, CMT grade II and aggressive tumor) in objective of 40×. **(A)** Tubular adenoma in objective of 40× (HE). **(B)** Tubular adenoma in objective of 40× (GATA-3). **(C)** Tubular adenoma in objective of 40× (ER). **(D)** Tubular adenoma in objective of 40× (Ki-67). **(E)** CMT grade I in objective of 40× (HE). **(F)** CMT grade I in objective of 40× (GATA-3). **(G)** CMT grade I in objective of 40× (ER). **(H)** CMT grade I in objective of 40× (Ki-67). **(I)** CMT grade II in objective of 40× (HE). **(J)** CMT grade II in objective of 40× (GATA-3). **(K)** CMT grade II in objective of 40× (ER). **(L)** CMT grade II in objective of 40× (Ki-67). **(M)** Solid carcinoma in objective of 40× (HE). **(N)** Solid carcinoma in objective of 40× (GATA-3). **(O)** Solid carcinoma in objective of 40× (ER). **(P)** Solid carcinoma in objective of 40× (Ki-67).

**Figure 2:**
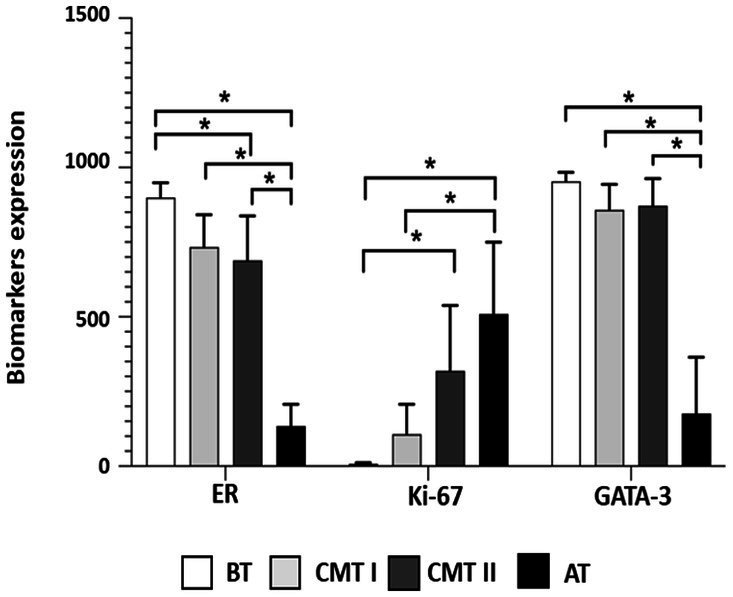
Graphic representation of the expression of biomarkers in samples of canine mammary tumors. Brackets with asterisks indicate significant differences between the corresponding groups. Data were analyzed using the Kruskal–Wallis test, followed by the Dunn’s test. Statistical significance: (^*^) *p* < 0.05, (^**^) *p* < 0.01, and (^***^) *p* < 0.0001. G-BT, group of benign tumors; G-CMT-I, group carcinomas in a mixed tumor (CMT) grade I; G-CMT-II, group of CMT grade II; G-AT/CMT-III, group of aggressive tumors and CMT grade III.

Determination of ER, Ki-67 and GATA-3 expression frequency by counting 1,000 cells per case after immunohistochemical marking (Figure 1), was analyzed and compared between groups G-BT, G-CMT-I, G-CMT-II, and G-AT/CMT-III (Table 2). There was higher ER expression in well-differentiated tumors, and lower ER expression in aggressive tumors (*p* < 0.05) (Figures 1, 2). There was higher expression of Ki-67 in aggressive tumors, while in benign neoplasms Ki-67 expression was significantly lower (*p* < 0.05) (Figures 1, 2). GATA-3 immunostaining frequency was equivalent to that of ER: the degree of GATA-3 expression in tumors well differentiated was significantly higher in the group of benign tumors and well-differentiated carcinomas, while its expression was lower in aggressive tumors (*p* < 0.05) (Figure 2 and Table 2).

**Table 2:** Marker expression in canine mammary tumor according to the analyzed group in percentage (%).

	ER	GATA-3	Ki-67
G-BT	89.93 ± 4.95a	95.38 ± 3.03a	0.71 ± 0.38a
G-CMT-I	73.40 ± 10.74b	85.77 ± 8.54b	10.71 ± 9.97a
G-CMT-II	68.87 ± 14.93b	87.18 ± 9.12b	31.87 ± 21.80b
G-AT/CMT-III	13.38 ± 7.36c	17.62 ± 18.85c	50.89 ± 24.08b

Different letters in the column indicate a difference in the expression of the marker between groups. Significant differences (*p* < 0.05). G-BT, group of benign tumors; G-CMT-I, group carcinomas in a mixed tumor (CMT) grade I; G-CMT-II, group of CMT grade II; G-AT/CMT-III, group of aggressive tumors and CMT grade III.

There was a direct correlation between GATA-3 expression and ER expression, ranging from strong to very strong, in the G-CMT-I, G-CMT-II, and G-AT/CMT-III groups (*p* < 0.05) (Table 3). At the same time, there was an inverse correlation between the Ki-67 proliferation index and GATA-3 expression in all groups, which ranged from moderate to very strong (*p* < 0.05) (Figure 2 and Table 3).

**Table 3:** Correlation of the GATA-3 with the ER and Ki-67 biomarkers.

	GATA-3	Spearman *r*	Value of *p*
ER	G-BT	0.7622	0.0132
	G-CMT-I	0.9477	0.0001
	G-CMT-II	0.8303	0.0047
	G-AT/CMT-III	0.9362	0.0002
Ki-67	G-BT	−0.6626	0.0413
	G-CMT-I	−0.7455	0.0174
	G-CMT-II	−0.9329	0.0002
	G-AT/CMT-III	−0.8875	0.0012

Relationship of correlation coefficients and value of the degree of significance. The correlation of Spearman analyzed all data. Bold = significant correlations (*p* < 0.05). G-BT, group of benign tumors; G-CMT-I, group carcinomas in a mixed tumor (CMT) grade I; G-CMT-II, group of CMT grade II; G-AT/CMT-III, group of aggressive tumors and CMT grade III.

### 3.3. Comparison of survival curves

The shortest survival time after surgery of 30 days occurred with a dog from the aggressive tumor group. This dog developed inflammatory mammary carcinoma secondary to surgery. A necropsy could not be performed on this animal to identify possible sites of metastasis, as the owner did not authorize the procedure. The imaging exams performed before the mastectomy to determine the clinical staging of the patient by TNM system did not indicate or suggest pulmonary metastasis in the animals, regardless of the group. However, the clinical records of some animals at the survival follow-up informed about the possible cause of death as respiratory failure for pulmonary metastasis. Unfortunately, there was no case with information about necropsy or histopathological analysis of the lung fragments. Thus, stage V was not considered in this study.

**Figure 3:**
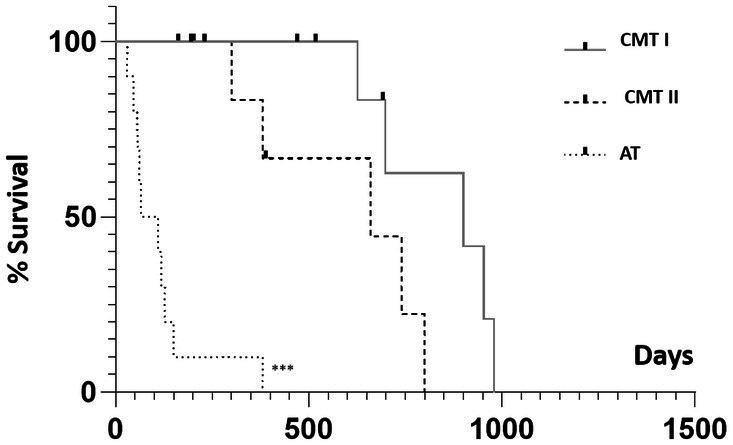
Kaplan–Meier survival curve according to the group studied (G-CMT-I, G-CMT-II and G-AT/CMT-III), regardless of GATA-3 expression. (^***^) express a significant statistical difference among the curves by the log-rank test with *p* < 0.001. G-CMT-I, group carcinomas in a mixed tumor (CMT) grade I; G-CMT-II, group of CMT grade II; G-AT/CMT-III, group of aggressive tumors and CMT grade III.

The longest survival time after surgery was 1,007 days and involved a dog from the benign tumor group. At the end of this study, all G-BT animals survived (*n* = 10/10; 100%), whereas all G-AT/CMT-III dogs died (*n* = 10/10; 100%). The survival analysis was restricted to the 30 female dogs with malignant mammary tumors. The median survival time was 900, 660, and 88 days for the G-CMT-I, G-CMT-II, and G-AT/CMT-III groups (*p* < 0.0001) (Figure 3). There were significant differences in post-surgery survival times between groups G-CMT-I and G-AT/CMT-III (*p* < 0.001, HR 7.002 and 95% CI 2.073–23.66); and G-CMT-II and G-AT/CMT-III (*p* < 0.001, HR 6.113 and CI 1.889–19.78). However, there was no difference in post-surgery survival times between G = CMT-I and G-CMT-II (*p* = 0.0611, HR 2.992 and CI 95% CI 0.7157–12.51) (Figure 3).

The expression of GATA-3 in neoplastic epithelial cells was considered high when the percentage of GATA-3 cells marked in a total of 1,000 cells was ≥79.4%; and low when positive cells for GATA-3 was <79.4%. The survival curves were stratified based on the expression of GATA-3 and analyzed only in malignant tumours. The results indicated that dogs with tumours with a high GATA-3 expression also had a significantly higher survival rate than those with low GATA-3 expression (*p* < 0.0001, HR 5.021 and 95% CI 1.741–14.48), and presented a median survival of 740 days. The highest death rate (*n* = 11/13, 84,61%) occurred among dogs that presented tumors with low GATA-3 expression, with a median survival of 119 days. Data were analyzed using the Kaplan–Meier method, and there was a statistically significant difference between the GATA-3 intervals (*p* < 0.001).

**Figure 4:**
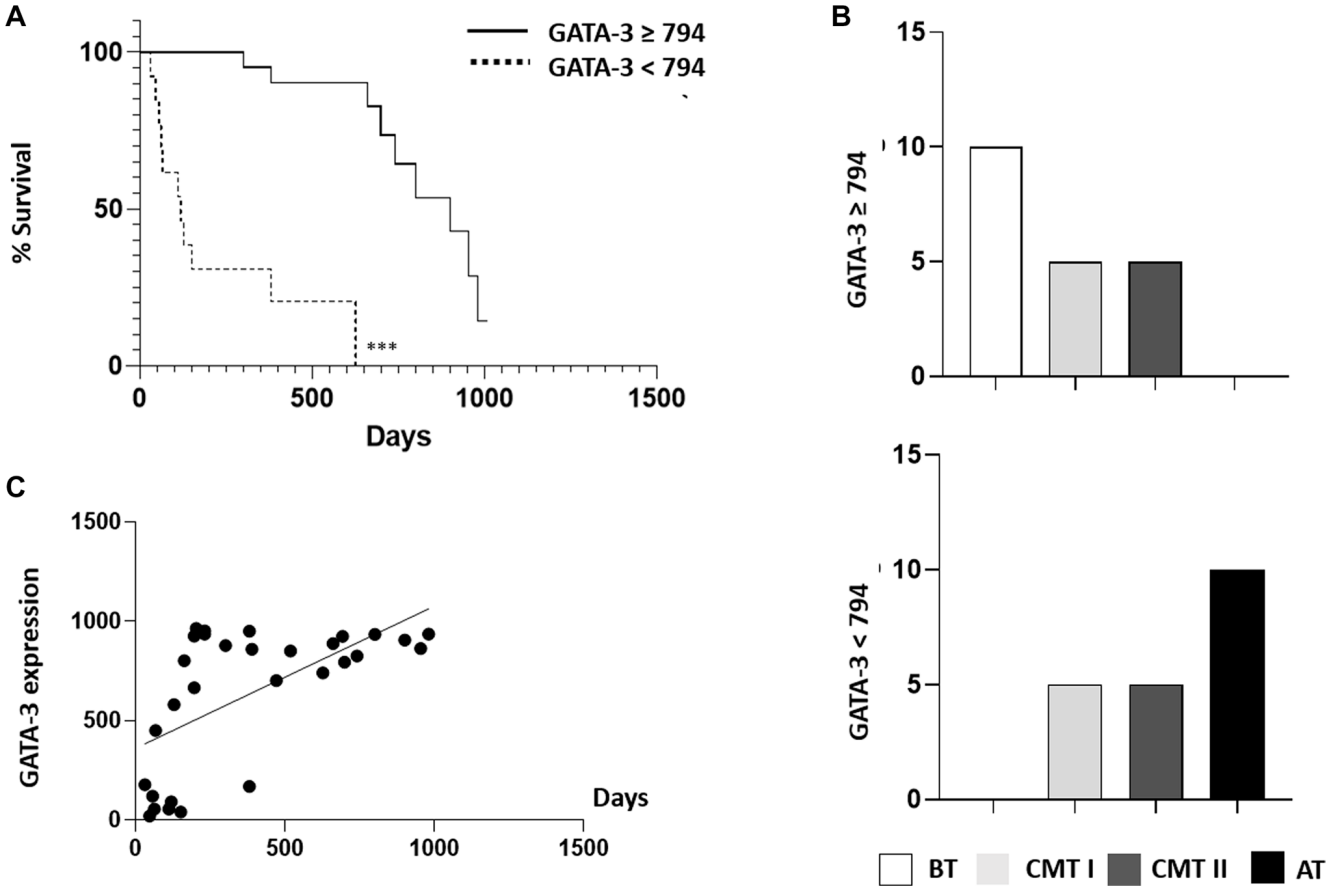
Relationship between the survival rate of canine mammary tumors and the expression of the biomarker GATA-3. **(A)** Kaplan–Meier survival curve for all animals with malign mammary tumors (G-CMT-I, G-CMT-II and G-AT/CMT-III) characterized according to the intensity of GATA-3 expression (high ≥79.4% and low <79.4%) of GATA-3-stained cells. (^***^) express a significant statistical difference among the curves by the log-rank test with *p* < 0.001. **(B)** Graphs represent the relationship of GATA-3 categorized according to the frequency of cases with high (≥79.4%) and low (<79.4%) expression intensity in the different types of canine mammary tumors (BT, CMT I, CMT II, and AT). **(C)** Correlation graph between GATA-3 expression and survival of animals with malignant mammary tumors. The correlation coefficient was positive, proving to be a directly proportional relationship. Data were obtained by Pearson’s correlation test. Degree of significance: *p* < 0.0001. G-BT, group of benign tumors; G-CMT-I, group carcinomas in a mixed tumor (CMT) grade I; G-CMT-II, group of CMT grade II; G-AT/CMT-III, group of aggressive tumors and CMT grade III.

When GATA-3 expression (high ≥79.4% vs. low <79.4%) was analyzed in accordance with histological group, the benign tumor group showed a greater number of samples with high expression of GATA-3 (Figures 4A–D). Comparatively, the group of dogs with aggressive tumors revealed higher frequencies of samples with low GATA-3 expression and lower survival rates (Figure 4B). By correlating GATA-3 expression with survival in the cases of malignant mammary tumors, there was a directly proportional relationship and moderate and significant correlation (*p* = 0.0003; *r* = 0.6127) (Figure 4C).

### 3.4. Performance indices of GATA-3

**Figure 5:**
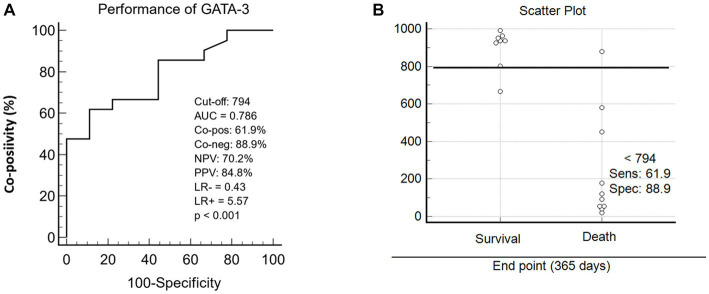
Graphical representation of cut-off performance indices of GATA-3 expression. **(A)** Scatter plot between GATA-3 expression and disease outcome in 365 days after mastectomy in animals with malignant mammary tumors. **(B)** ROC (Receiver Operating Characteristic) curve indices, using a specific cut-off point, including: area under curve/overall accuracy (AUC), Co-positivity (Co-pos), Co-negativity (Co-neg), negative and positive predictive values (NPV and PPV) and relative risk/likelihood ratio (LR^−^ and LR^+^).

Scatter plot analysis revealed that the cut-off point (≥79.4%cells GATA-3 positive) highlighted statistically significant differences and defined the disease progression outcomes toward survival or death in dogs with mammary neoplasms. Analysis of performance indices showed an outstanding global accuracy value (AUC = 0.786), negative predictive value (NPV = 70.2%), and an LR = −0.43 (Figure 5A). In addition to these data, the cut-off provided a specificity of 88.9% and a sensitivity of 61.9% (Figure 5B). The analyses considered the canine patients’ status (alive/dead) at 365 days post-mastectomy.

The analysis of the Kaplan–Meier survival curves further validated the previous findings, demonstrating that dogs with GATA-3 ≥ 79.4% had a significantly greater survival rate than those with lower expressions of this factor (*p* < 0.001, HR 7.901 and CI 2.182–28.61). GATA-3 was shown to be a biomarker of survival in dogs with malignant tumors (30), with 50.0% (8/16) of those that had a high GATA-3 expression (≥79.4%) surviving, while only 14.28% (2/14) of those with low expression (<794) survived, as shown in Figure 4B. The highest number of deaths (*n* = 12/14; 85,71%) occurred among dogs that presented tumors with GATA-3 < 79.4%.

### 3.5. Univariate and multivariate analysis

GATA-3 (*p* < 0.001), tumor size (*p* < 0.001), lymph node (LN) metastasis (*p* < 0.001), clinical stage (*p* = 0.002), histological grade (*p* < 0.001) and Ki-67 (*p* < 0.003) displayed significant associations with survival. The multivariate analysis revealed that only GATA-3 (*p* = 0.015) remained as an independent prognostic factor of mortality in the final model (Table 4). The ER showed significance only in the multivariate analysis (*p* = 0.005) (Table 4). Additional analysis demonstrated a significant association between tumor size (*p* < 0,001), lymph node (LN) metastasis (*p* = 0.004), clinical stage (*p* = 0.016), histological grade (*p* = 0.006), and survival (*p* < 0.0001), as well as distinct intervals of GATA-3 expression (Table 5).

**Table 4:** Associations between survival and clinical-pathological parameters of female dogs with mammary tumors analyzed by univariate and multivariate methods.

Parameters	Univariate^a^		Multivariate^b^	
	Log-rank (HR)	Value of *p*	Hazard ratios	Value of *p*
	(IC 95%)		(IC 95%)	
GATA-3	0.131 (0.03–0.443)	<0.001^*^	8.751 (1.524–50.261)	0.015^*^
Tumor size	0.158 (0.054–0.463)	<0.001^*^	1.544 (0.360–6.633)	0.559
LN metastasis	0.110 (0.024–0.497)	<0.001^*^	7.105 (0.599–84.316)	0.120
Clinical stage	0.289 (0.124–0.671)	0.002^*^	2.963 (0.379–23.138)	0.300
Histological grade	0.147 (0.022–0.961)	<0.001^*^	1.283 (0.353–4.670)	0.705
ER	0.131 (0.014–1.19)	0.068	8.013 (1.885–34.066)	0.005^*^
Ki-67	0.093 (0.038–0.225)	0.003^*^	3.635 (0.763–18.97)	0.068

^*^Significant differences (*p* < 0.005), A, log-rank; B, cox regression; LN, lymph node. ER, estrogen receptor; Ki-67, proliferation index.

**Table 5:** Association between clinical-pathological parameters and interval of GATA-3 (≥79.4% positive cells).

Parameters		GATA-3		*p*	(Odds ratio, 95%CI)
		<79.4%	≥79.4%		
Tumor size	<5 cm	2	23	<0.001	9.28 (3.07–19.76)
	≥5 cm	11	4		
LN metastasis	No	5	15	0.004^*^	12.02 (1.88–36.76)
	Yes	8	2		
Clinical stage	I–II	1	9	0.016^*^	19.08 (5.64–26.48)
	III–IV	12	8		
Biological behavior	Benign	0	10	^**^	^**^
	Malignant	13	7		
Histological grade	I/II	7	17	0.006^*^	9,63 (6.58–20.52)
	III	6	0		
Survival	Alive	1	18	<0.001^*^	24.04 (2.68–114.72)
	Dead	12	9		
Ki-67	<14%	0	9	^**^	^**^
	≥14%	13	18		
ER	<10%	3	0	^**^	^**^
	≥10%	10	27		

Selected by univariate and multivariate analyzes. For the univariate analysis the correlation of Spearman was used to verify between variables. For multivariate analysis used logistics regression. Significant differences for *p* < 0.05 was highlighted by asterisk. LN, lymph node; ER, estrogen receptor; Ki-67, proliferation index.

## 4. Discussion

The present study evaluated GATA-3 expression in spontaneous mammary tumors of female dogs in accordance with degree of malignancy, as defined by tumor histopathological and clinical classification, and the relationship between this expression and survival rates. GATA-3 has been reported as a sensitive and specific marker for diagnosing human breast carcinomas, and high expression of this factor is associated with favorable prognostic outcomes (7, 41–45). However, no study had evaluated the prognostic value of GATA-3 protein expression in mammary tumors of female dogs, which are commonly used as animal models in human breast cancer studies.

We found expression of GATA-3 in an exclusive nuclear location of epithelial cells of canine mammary tumors. Moreover, similar to mammary tumors in women, this study showed that GATA-3 expression was inversely proportional to the malignancy potential of mammary tumors in dogs. Canine benign tumors or well-differentiated carcinomas exhibited intense and diffuse GATA-3 staining patterns of by immunohistochemistry, while canine aggressive tumors showed only mild and scattered results or the absence of stain. Our findings in the present study on canine mammary tumors correlated to research findings in human counterparts. Gentile et al. (28) reported low levels of GATA-3 in cells of complex adenoma and simple carcinoma, and high levels among complex and mixed carcinomas in their *in vitro* study, which are divergent from those reported in our study. However, these results highlight the possible influence of the tumor microenvironment and histopathological grading on the expression profile and behavior of different molecules, such as GATA-3.

The results of the present study confirm the correlation between the most significant expression of GATA-3 and cell differentiation, given its overexpression in benign tumors and well-differentiated carcinomas. In women, the degree of tumoral differentiation may result from the interaction of GATA-3 with the BRCA1 gene, which cause the formation of protein complexes that suppress genes associated with the triple negative basal-like phenotype (ER^−^, PR^−^, HER-2^−^, and CK 5/6^+^ or CK14^+^) in breast carcinomas (45). In addition, differentiation may result from the interaction of GATA-3 with microRNA-29b, which promotes alterations with the tumor microenvironment and inhibits metastasis by interfering with angiogenesis and extracellular matrix regulation through collagen remodeling and proteolysis (15). Additionally, GATA-3 prevents epithelial-mesenchymal transition, which can justify its greater expression in well-differentiated tumors (13, 14). GATA-3 is associated with luminal cell differentiation in mammary glands (43, 46), and the majority of human breast tumors originate from epithelial luminal cells (10, 18). In women with well-differentiated luminal A tumors, GATA-3 expression is high, while its lower expression is reported in triple-negative breast carcinomas, which are more aggressive (41, 43, 47).

Furthermore, a sequencing study by Cohen et al. (2014) demonstrated frequent somatic mutations in GATA-3 in patients with luminal breast cancer, and that mutations occurred mostly within the DNA-binding domain of GATA-3 and were capable of modulating its activity (20). The authors showed that there was an inability of mutant GATA-3 to activate the IL5 promoter, suggesting that mutGATA-3 might have a distinct effect on a subset of promoters where GATA-3 is required to recruit other transcription factors (20). It was also showed that mutGATA-3 had a weaker activity or failed to regulate GATA-3 target genes, concomitantly with altered activity (20). These observations suggest that the mutated GATA-3 protein may lead to disparate mechanisms associated with cancer.

The results of the present study encourage other studies to evaluate this transcription factor in different molecular subtypes of dog mammary tumors, especially those of ER^−^ tumors. In a previous study, our group found a high expression of GATA-3 in triple negative mammary tumors surgically removed from two male dogs (27), which raised the hypotheses that sex may influence GATA-3 expression. Indeed, similar results have been reported in human male breast tumors, whereby GATA-3 was less frequently expressed and uncorrelated with hormone receptors (ER/PR), distant metastases, or survival rate (41). This hypothesis is reinforced by the low expression of GATA-3 in tumors with a triple negative phenotype and the correlation of this factor with an unfavorable prognosis in women (44, 48).

GATA-3 is crucial for the regulation of the function of the ER (44). In the present study, ER expression was proportional to tumor differentiation, with an expected high expression in benign tumors and well-differentiated carcinomas and low or absent expression in aggressive tumors. The expression of hormone receptors, particularly the ER, has a direct relationship with the biological behavior of the tumor and the degree of differentiation of breast neoplasms (49, 50). Exceptions to this are micropapillary carcinomas, in which ER expression is not related to reduced aggression (51).

The results obtained in this study indicate a positive correlation between ER and GATA-3 expression in mammary tumors in dogs. Similarly, immunohistochemical expression of GATA-3 in human breast cancer showed a direct correlation with that of ER, as the marker was related to the ER^−^ phenotype, which is characteristic of poorly-differentiated aggressive tumors (43, 48, 52). The intensity of the correlation between ER and GATA-3 expression in this study was high, with a coefficient ranging from 0.76 to 0.94, which was compared to the strong coefficient of correlation found in a study on tumors in women, which ranged from 0.9 to 0.96 (40).

The expression of the Ki-67 proliferation marker in relation to that of GATA-3 expression in the dog groups studied in this work showed a strong negative correlation. The lowest expression of GATA-3 was observed in tumors with high rates of proliferation and aggressive characteristics. These results were similar to those described in the literature regarding breast tumors in women. Previous studies have reported the greater expression of Ki-67 in malignant human breast tumors, especially in less differentiated cases, as well as an inverse correlation between Ki-67 and ER expression (51, 53). Ki-67 is defined as an unfavorable prognostic marker; its high nuclear expression is related to the differentiation degree, histopathological classification, and metastatic potential of the neoplasm (54). Because GATA-3 actively participates in luminal cell differentiation, its low expression correlates with higher proliferative indices and the development of less differentiated tumors (47, 54).

In this study, a positive and significant correlation was observed between GATA-3 expression and survival. Moreover, the performance indices demonstrated that GATA-3 is an excellent survival biomarker in terms of the outcome of canine mammary neoplasia. Higher indices of GATA-3 expression were found to be significantly related to improved clinical-pathological parameters, smaller tumor sizes, lower grades, and higher survival rates. GATA-3 expression has been shown as a marker of good prognosis in human breast cancer and its higher expression was consistent with better rates of recurrence-free and overall survival (52).

In the present study, pulmonary metastasis was not identified in any of the dogs until the moment of mastectomy when the tumors fragments were collected for the GATA-3 expression analysis. The total number of cases that showed nodal metastasis, the clear majority displayed low GATA-3 expression. In addition, low GATA-3 expression was related to lower median survival when compared to animals with high GATA-3 expression. Low GATA-3 expression is a significant risk factor for death in women with breast cancer (14, 43). In a study using aggressive breast cancer cell lines in mice susceptible to lung metastasis, an increased expression of GATA-3 resulted in reduced tumor growth and a lower rate of lung metastasis (15). Those authors stated that the expression of GATA-3 may inhibit the expansion of neoplastic cells within the lung parenchyma through gene regulation mechanisms.

The predictive potential of GATA-3 in women with breast tumors has been explored (14, 43, 55). A low expression of this factor implies an unfavorable prognosis (48) and non-responsiveness to hormone therapy even in ER-positive tumors (56). However, Voduc et al. (57) did not identify a correlation between GATA-3 expression and prognostic factors using multivariate analysis. The use of semi-quantitative or qualitative methods to assess GATA-3 expression may contribute to the divergence of results in terms of the relationship between prognostic factors and GATA-3 in breast cancer in women. Due to this divergence, we established a cut-off point for GATA-3 immunohistochemistry analysis of 794 labeled cells/1000 cells or 79.4% labeled cells and demonstrated that this ratio was effective by analyzing it using the ROC curve, performance index, and likelihood ratio.

We recognize the limitations of our study, including the small sample size and the absence of molecular phenotyping of the tumors. This precluded further comparison with the results observed with women, mainly in relation to triple negative tumors. However, we believe that the objective of our study was achieved by demonstrating the prognostic importance of GATA-3 based on the strong correlation with ER and Ki-67 and its association with important prognostic factors such as tumor size, histopathological grade, and increased survival in canine mammary neoplasms. These results allow for further exploration regarding the role of GATA-3 in tumor progression and diagnosis and the response to therapy of mammary tumors in both species.

The results obtained in this study indicated that GATA-3 expression was higher in benign tumors and well-differentiated carcinomas, showing a positive correlation with ER and a negative correlation with the Ki-67 proliferation marker. The high expression of GATA-3 was associated with a higher survival rate, and can be considered an independent prognostic factor in mammary neoplasms of female dogs. Our results were similar to reported descriptions of these markers breast cancers of women. The chosen cut-off point proposed for the evaluation of GATA-3 allowed for the discernment of female dogs with higher probabilities of survival. These data promote the use of GATA-3 in future studies of tumor progression, and as a prognostic and monitoring biomarker in dogs with mammary carcinomas.

## Data availability statement

The original contributions presented in the study are included in the article/supplementary material, further inquiries can be directed to the corresponding author.

## Ethics statement

The animal study was reviewed and approved by Ethics Committee on the Use of Animals of the School of Veterinary Medicine of the Federal University of Bahia (protocol no. 17/2021). Written informed consent was obtained from the owners for the participation of their animals in this study.

## Author contributions

AE-L, KD, and GD-G contributed to the conception and design of the study. GD-G, CC, and LS conducted the fieldwork. GD-G, CC, KD, GC, and AE-L conducted the laboratory work. GD-G and AE-L organized the database. GD-G, LR, and AE-L performed the statistical analysis. GD-G, AH-B, VS, LR, AE-L, and SB-M wrote the manuscript. AE-L was responsible for fund acquisition. All authors contributed to the article and approved the submitted version.

## Funding

We thank the Coordenação de Aperfeiçoamento de Pessoal de Nível Superior for the Scholarship to AE-L (CAPES/PRINT-Finance Code 001: no. 88887.694500/2022-00) and GC is supported by a grant from Fundação de Amparo à Pesquisa do Estado de Minas Gerais (FAPEMIG) [Rede Mineira de Pesquisa Translacional em Imunobiológicos e Biofármacos no Câncer (REMITRIBIC, RED-00031-21)]. AE-L (Proc. 310248/2021-3), SB-M (Proc. 312022/2021-2), and GC (Proc. 303368/2021-7) are supported by research productivity and technological development fellowships from the National Council for Scientific and Technological Development (CNPq).

## In memoriam

We dedicate this study to our colleague Dr. Emanoel Martins-Filho while recognizing his dedication and ethics in research and life. Dr. Emanoel Martins-Filho passed away before submitting the final version of this manuscript and was a victim of the COVID-19 pandemic.

## Conflict of interest

The authors declare that the research was conducted in the absence of any commercial or financial relationships that could be construed as a potential conflict of interest.

## Publisher’s note

All claims expressed in this article are solely those of the authors and do not necessarily represent those of their affiliated organizations, or those of the publisher, the editors and the reviewers. Any product that may be evaluated in this article, or claim that may be made by its manufacturer, is not guaranteed or endorsed by the publisher.
